# Association between platelet count and mucosal healing in Japanese patients with ulcerative colitis: a cross-sectional study

**DOI:** 10.1186/s12876-020-01538-y

**Published:** 2020-11-16

**Authors:** Shinya Furukawa, Sen Yagi, Kana Shiraishi, Kenichirou Mori, Tomoyuki Ninomiya, Keitarou Kawasaki, Yuji Mizukami, Seiyuu Suzuki, Masayoshi Uraoka, Naozumi Shibata, Sanae Nakamura, Satoshi Imamine, Hidehiro Murakami, Katsuhisa Ohashi, Masamoto Torisu, Aki Hasebe, Harumi Yano, Masato Murakami, Eiji Takeshita, Yoshio Ikeda, Yoichi Hiasa

**Affiliations:** 1 Health Services Center, Ehime University, Bunkyo,Matsuyama, Ehime 790-8577 Japan; 2 Department of Internal Medicine, Saiseikai Matsuyama Hospital, Matsuyama, Ehime 791-8026 Japan; 3 Department of Gastroenterology and Metabology, Ehime University Graduate School of Medicine, Toon, Ehime 791-0205 Japan; 4 Department of Gastroenterology, Ehime Prefectural Central Hospital, Matsuyama, Ehime 790-0024 Japan; 5 Department of Internal Medicine, Saiseikai Imabari Hospital, Imabari, Ehime 799-1592 Japan; 6 Department of Gastroenterology, Matsuyama Shimin Hospital, Matsuyama, Ehime 790-0067 Japan; 7 Department of Gastroenterology, Sumitomo Besshi Hospital, Niihama, Ehime 792-8543 Japan; 8 Uraoka Gastrointestinal Clinic, Matsuyama, Ehime 790-0852 Japan; 9 Department of Gastroenterology, Ehime Prefectural Niihama Hospital, Niihama, Ehime 792-0042 Japan; 10 Department of Gastroenterology, NTT Matsuyama Hospital, Matsuyama, Ehime 790-0802 Japan; 11 Department of Internal Medicine, Shiritsu Oozu Hospital, Oozu, Ehime 795-0013 Japan; 12 Ohashi Clinic Participating in Gastro-Enterology and Ano-Proctology, Niihama, Ehime 792-0856 Japan; 13 Department of Internal Medicine, Saiseikai Saijo Hospital, Saijo, Ehime 793-00257 Japan; 14 Department of Gastroenterology, Shikoku Cancer Center, Matsuyama, Ehime 791-0280 Japan; 15 Department of Gastroenterology, Saijo City Shuso Hospital, Saijo, Ehime 793-0027 Japan; 16 Department of Gastroenterology, Murakamikinen Hospital, Saijo, Ehime 793-0030 Japan; 17 Department of Inflammatory Bowel Diseases and Therapeutics, Ehime University Graduate School of Medicine, Matsuyama, Ehime 791-0205 Japan; 18 Endoscopy Center, Ehime University Hospital, Toon, Ehime 791-0205 Japan

**Keywords:** Platelet, Mucosal healing, Ulcerative colitis

## Abstract

**Background:**

Mucosal healing (MH) has been indicated as the therapeutic goal for ulcerative colitis (UC). Platelet count is known as an inflammation evaluation. However, the association between platelet count and MH among patients with UC is still scarce. We therefore assessed this issue among Japanese patients with UC.

**Methods:**

The study subjects consisted of 345 Japanese patients with UC. Platelet count was divided into quartiles on the basis of the distribution of all study subjects (low, moderate, high, and very high). Several endoscope specialists were responsible for evaluating MH and partial MH, which was defined as a Mayo endoscopic subscore of 0 and 0–1, respectively. Estimations of crude odds ratios (ORs) and their 95% confidence intervals (CIs) for partial MH and MH in relation to platelet count were performed using logistic regression analysis. Age, sex, CRP, steroid use, and anti-Tumor necrosis factor α (TNFα) preparation were selected a priori as potential confounding factors.

**Results:**

The percentage of partial MH and MH were 63.2 and 26.1%, respectively. Moderate and very high was independently inversely associated with partial MH (moderate: OR 0.40 [95%CI 0.19–0.810], very high: OR 0.37 [95%CI 0.17–0.77], p for trend = 0.034). Similarly, moderate, high, and very high were independently inversely associated with MH (moderate: OR 0.37 [95% CI 0.18–0.73], high: OR 0.41 [95% CI 0.19–0.83], and very high: OR 0.45 [95% CI 0.21–0.94], p for trend = 0.033) after adjustment for confounding factors.

**Conclusions:**

Among patients with UC, platelet count was independently inversely associated with MH

## Background

Ulcerative colitis (UC) is a chronic inflammatory bowel disease (IBD) characterized by a disease course involving relapses and remissions [1]. Mucosal healing (MH) is inversely associated with clinical relapse, rates of hospitalization and surgery, and incidence of colorectal cancer [2–7]. Thus, MH has been indicated as the therapeutic goal for UC. Endoscopy is considered the gold standard for evaluating MH, but frequent endoscopic procedures are invasive, inconvenient, expensive, and occasionally cause complications.

Platelet count is considered to be an inflammation marker. Platelet count among active UC patients was higher than that among inactive UC patients [8, 9]. Platelet count among patients with complete MH was lower than that among patients with partial MH [10]. Two studies identified a positive relationship between platelet count and the severity of endoscopic severity among patients with UC [11, 12]. Elevated platelet count was significantly positively associated with relapse among patients with UC [13]. In a Spanish study of patients with IBD, however, platelet count was not associated with endoscopic activity among patients with UC [14]. The association between platelet count and MH among patients with UC is still scarce.

Thus, we aimed to evaluate the association between platelet count and MH among Japanese patients with UC.

## Material and methods

### Study population

The study subjects consisted of 387 Japanese patients with UC at the Department of Gastroenterology and Metabology at the Ehime University Graduate School of Medicine, and at several affiliated hospital in Ehime prefecture (the Ehime Clinical Network for Alimentary Diseases [ECNAD] study group). All patients were diagnosed with UC based on endoscopic, radiological, histological, and clinical criteria. After 42 patients were excluded due to incomplete data, the final analysis sample in this study consisted of 345 patients assessed for endoscopy examination, medication, and platelet count. The study protocol conforms to the ethical guidelines of the 1975 Declaration of Helsinki (6th revision, 2008) and was approved by the institutional review board of the Ehime University Graduate School of Medicine (1,505,011). Consent was obtained mainly for outpatients at each hospital, as well as inpatients for ulcerative colitis. The study was explained and enrolled to all those diagnosed with ulcerative colitis. Well-trained staff obtained written informed consent from all patients enrolled. This study analyzed group was registered during the period from 2015 to 2019.

### Measurements

Information on endoscopic findings, use of drugs for UC, and platelet count and CRP were collected using medical records. Platelet count was determined from routine blood levels obtained from patient files at the time the patients were included in the study. Blood samples were taken the morning after overnight fasting. Blood examination was collected when a colonoscopy is reserved or when the colonoscopy examination is performed. There could be a time difference of about two months.

### Definition of mucosal healing

We evaluated mucosal status by total colonoscopy. The Mayo endoscopic subscore (MES) contains the following four categories [15]: 0, normal or inactive disease; (1) mild disease with erythema, decreased vascular patterns, and mild friability; (2) moderate disease with marked erythema, absence of vascular patterns, friability, and erosions; (3) severe disease with spontaneous bleeding and ulceration. Partial MH and MH were defined as category 0 and 0–1 in this study, respectively. Several endoscope specialists were responsible for evaluating MES and MH, and all endoscopists were blinded to platelet count.

### Statistical analysis

Platelet count was divided into quartiles on the basis of the distribution of all study subjects. Platelet count was classified into four categories: (1) low platelet, < 20.4(× 10^4^/μL) (reference); (2) moderate platelet, 20.4–25.0 (× 10^4^/μL); (3) high platelet, 25.0–29.6 (× 10^4^/μL), and (4) very high platelet, > 29.6 (× 10^4^/μL). Estimations of crude odds ratios (ORs) and their 95% confidence intervals (CIs) for partial MH and MH in relation to platelet count were performed using logistic regression analysis. Multiple logistic regression analyses were used to adjust for potential confounding factors. Age, sex, CRP, steroid use, and anti-TNFα preparation were selected a priori as potential confounding factors. A receiver operating characteristic (ROC) curve was generated, and the area under the curve was calculated to show the utility of the platelet count in indicating mucosal healing. We used Youden’s index to calculate the cutoff value of platelets. Statistical analyses mainly were performed using SAS software package version 9.4 (SAS Institute Inc., Cary, NC, USA). The ROC curve, cutoff value of the platelet count, sensitivity, and specificity were analyzed using JMP 14.2 software (SAS Institute Inc., Cary, NC, USA). All probability values for statistical tests were two-tailed, and P < 0.05 was considered statistically significant.

## Results

**Fig. 1:**
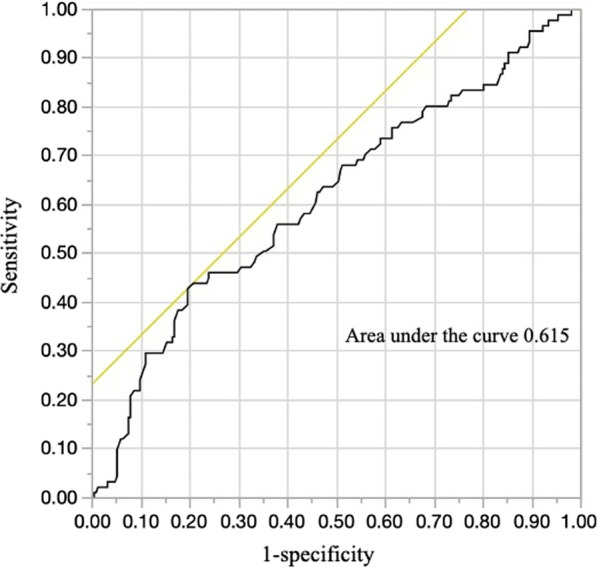
Receiver operating characteristic curve of platelet count for identifying mucosal healing based on the Mayo endoscopic subscore. The receiver operating characteristic (ROC) curve platelet count for identifying mucosal healing had an area under the curve of 0.613. When the cutoff value of the platelet count was 20.4, the sensitivity and specificity were 42.9% and 76.7%, respectively

Table 1 shows the characteristics of the 345 study participants. The mean age was 50.2 years, and the percentage of male patients was 59.7%. Use of 5-aminoasalicylates, prednisolone, azathioprine, and TNF-α monoclonal antibody preparations were reported to be 91.0%, 20.6%, 15.4% and 5.5%, respectively. The percentage of partial MH and MH were 63.2% and 26.1%, and the mean CRP and platelet count were 0.39 ± 1.13 (mg/dl) and 25.77 ± 7.80 (× 10^4^/μL). The ROC curve of the platelet count for identifying mucosal healing had an area under the curve of 0.615 (Fig. 1).

**Table 1:** Clinical characteristics of the 345 study participants

Variable	n (%)
Age, years, mean ± SD	50.2 ± 16.6
Male (%)	206 (59.7)
Disease extent (pancolitis/left-sided/procitis/others)	148/96/93/8
Medication	
5-aminoasalicylates (%)	314 (91.0)
Prednisolone (%)	71 (20.6)
Azathioprine	53 (15.4)
TNF-α monoclonal antibody (%)	19 (5.5)
Mayo endoscopic subscore, mean ± SD	1.17 ± 0.9
Partial mucosal healing (Mayo endoscopic subscore ≤ 1) (%)	218 (63.2)
Mucosal healing (Mayo endoscopic subscore < 1) (%)	90 (26.1)
CRP (mg/dl)	0.39 ± 1.13
Platelet (× 10^4^/μL), mean ± SD	25.91 ± 7.81

*SD* standard deviation, *TNF* tumor necrosis factor

Table 2 shows crude and adjusted ORs and 95% CIs for partial MH and MH in relation to platelet count. The percentages of partial MH and MH among low, moderate, high, and very high platelet patients were 78.2%, 60.5%, 62.9%, 51.2%, 43.7%, 20.9%, 19.8% and 19.8%, respectively.

**Table 2:** Crude and adjusted odds ratios and 95% confidence intervals for mucosal healing in relation to platelet count

Variable (× 10^4^/μL)	Prevalence (%)	Crude OR (95% CI)	Adjusted OR (95% CI)
Partial MH (MES ≤ 1)			
Platelet ≤ 20.4	68/87 (78.2)	1.00	1.00
20.4 < platelet ≤ 25.0	52/86 (60.5)	0.43 (0.22–0.83)	0.40 (0.19–0.80)
25.0 < platelet ≤ 29.6	54/86 (62.9)	0.47 (0.24–0.92)	0.54 (0.26–1.13)
29.6 < platelet	44/86 (51.2)	0.29 (0.15–0.56)	0.37 (0.17–0.77)
P for trend			0.034
MH (MES < 1)			
Platelet ≤ 20.4	38/87 (43.7)	1.00	1.00
20.4 < platelet ≤ 25.0	18/86 (20.9)	0.34 (0.17–0.66)	0.37 (0.18–0.73)
25.0 < platelet ≤ 29.6	17/86 (19.8)	0.32 (0.16–0.62)	0.41 (0.19–0.83)
29.6 < platelet	17/86 (19.8)	0.32 (0.16–0.62)	0.45 (0.21–0.94)
P for trend			0.033

*OR* odds ratio, *CI* confidence interval

Odds ratios were adjusted for age, sex, CRP, use of prednisolone, and use of TNF-α monoclonal antibody

After adjustment for age, sex, CRP, use of steroid, and use of TNF-α monoclonal antibody preparations, moderate, and very high platelet counts but not high platelet were independently inversely associated with partial MH (moderate: OR 0.40 [95% CI 0.19–0.80], and very high: OR 0.37 [95% CI 0.17–0.77], p for trend p = 0.034). Similarly, platelet count was independentlyinversely associated with MH (moderate: OR 0.37 [95% CI 0.18–0.73], high: OR 0.41 [95% CI 0.19–0.83], and very high: 0.45 [95% CI 0.21–0.94], p for trend = 0.033). In sensitive analysis, azathioprine was not associated with platelet count.

## Discussion

In the present study, platelet count was independently inversely associated with partial MH and MH among 345 Japanese patients with UC.

Platelet count is known as an inflammation marker. Several studies had showed the association between platelet count and outcome among patients with UC. Platelet count among active stage patients was higher than among those with inactive stage UC and healthy subjects in a Greek study of subjects including those with UC and Crohn’s disease [8] and in a Turkish study of subjects with active UC and inactive UC [9]. In a Japanese study of 68 patients with UC, platelet count was positively associated with endoscopic activity [11]. In a Swish study of 280 subjects including those with UC, a positive association between platelet count and endoscopic activity based on a modified Baron score among patients with UC was found [12]. In a retrospective cohort study of 183 Japanese UC patients with clinical remission, platelet count among patients with complete mucosal healing (Mayo endoscopic subscore 0) was lower than that among those with partial mucosal healing (Mayo endoscopic subscore1). Furthermore, the change of platelet count from baseline was significantly associated with a shift from complete to partial MH [10]. In a prospective observational study of Japanese UC patients with MH, a platelet count at baseline in the relapse group was significantly higher than that among those in the non-relapse group. Additionally, a platelet count at baseline was significantly associated with relapse among patients with UC after adjustment for several confounding factors [13]. In a Spanish study of 123 patients with UC, a positive relationship between fecal calprotectin level, which is a reliable marker for MH, and platelet count was found [16]. The finding in this study was consistent with these studies that showed the association between platelet count and favorable prognosis. On the other hand, however, a similar association between platelet count and endoscopic activity was reported among patients with Crohn’s disease but not among those with UC [14]. The discrepancies regarding platelet count and MH among patients with UC might be explained, at least in part, by differences in year, duration of UC, sample size, and confounding factors considered.

The underlying mechanism linking platelet count and MH among patients with UC remains unclear, but there are some biologically plausible options. Clusters of differentiation 154 (CD154) plays a central role in co-stimulation and regulation of the immune response via T cell priming and activation of CD40-expressing immune cells [17]. CD 154 expression was found on platelets [18], and mucosal inflammation might elevate platelet count via CD 154 expression.

The platelet-to-lymphocyte ratio (PLR) has emerged as a useful marker revealing shifts in platelet and lymphocyte counts due to acute inflammatory. PLR can be calculated easily from a routine blood test without additional cost. PLR has been reported to be a useful biomarker in the prognosis of various cancers [19]. Several pieces of evidence regarding the association between PLR and UC exist. In a Turkish study of 104 patients with UC and a Korean case control study of 144 subjects including 48 patients with UC and 96 healthy controls, PLR among patients with UC was higher than that in controls, regardless of mucosal remission. PLR among patients with endoscopic active UC was higher than that in patients with endoscopic remission UC [20]. Similarly, in a Korean study, PLR among patients with severe UC was higher than that in patients with mild to moderate UC [21]. In an Italian study of 88 UC patients who started anti-TNF monotherapy, PLR was a useful prognostic biomarker of mucosal healing [22]. Platelet count and PLR might be cost-effective and easy-to-validate parameters for optimal treatment modality. Our findings are consistent with those of previous reports regarding the association between platelets (including PLR) and UC.

To date, MH has been indicated as the therapeutic goal for IBD [3, 23, 24]. Endoscopy is necessary to evaluate MH, but repeated endoscopy is extremely burdensome for patients. Monitoring platelet count might be useful both for patients with UC and their physicians. However, further research is needed to confirm the association between platelet and MH in future.

Our study has a few limitations. First, this was a cross-sectional study; therefore, we cannot conclude that there is a causal relationship between platelet count and MH. Second, most of the patients in this cohort may have been receiving treatment for a considerable period of time since their physicians may have opted to attempt to control inflammation. The long duration of such treatment might have masked the association between platelet count and MH. Third, data regarding fecal calprotectin was not available in this cohort. Fourth, the time difference between the blood test and the endoscopy may have caused a misclassification. However, non-differential misclassification causes a bias of the odds ratio towards the null. Finally, the patients in the current study were not likely representative of Japanese patients with UC. Nevertheless, the median age, male/female ratio, and use of 5-aminoasalicylates, prednisolone, thiopurines, and biologics were similar in the present study (48.0, 59.7%, 91.0%, 20.6%, 15.4%, and 5.5%, respectively) and in a Japanese national study based on UC claims data in 2016 (44.0, 63.9%, 96.2%, 15.5%, 13.8%, and 9.0%, respectively).

## Conclusions

Among Japanese patients with UC, platelet count may be independently inversely associated with partial MH and MH. This study confirmed the positive association between platelet count and MH shown in previous retrospective studies and reinforced a positive dose–response association. Monitoring the platelet count may be useful in the assessment of early response to medication and when endoscopy cannot be repeated, such as in elderly and infant patients with UC.

## Data Availability

The datasets used and/or analyzed during the current study are available from the corresponding author on reasonable request.

## Abbreviations

MH: Mucosal healing
UC: Ulcerative colitis
TNF: Tumor necrosis factor
IBD: Inflammatory bowel disease
MES: Mayo endoscopic subscore
CD: Clusters of differentiation
PLR: Platelet-to-lymphocyte ratio
